# Spermine mediated improvements on stomatal features, growth, grain filling and yield of rice under differing water availability

**DOI:** 10.1038/s41598-021-89812-1

**Published:** 2021-05-21

**Authors:** Zulkarami Berahim, Deivaseeno Dorairaj, Mohamad Husni Omar, Halimi Mohd Saud, Mohd Razi Ismail

**Affiliations:** 1 Laboratory of Climate-Smart Food Crop Production, Institute of Tropical Agriculture and Food Security, Universiti Putra Malaysia, 43400 Serdang, Selangor Malaysia; 2 Department of Crop Science, Faculty of Agriculture, Universiti Putra Malaysia, 43400 Serdang, Selangor Malaysia; 3 Department of Agriculture Technology, Faculty of Agriculture, Universiti Putra Malaysia, 43400 Serdang, Selangor Malaysia

**Keywords:** Plant development, Plant hormones, Plant physiology, Plant stress responses

## Abstract

Rice which belongs to the grass family is vulnerable to water stress. As water resources get limited, the productivity of rice is affected especially in granaries located at drought prone areas. It would be even worse in granaries located in drought prone areas such as KADA that receives the lowest rainfall in Malaysia. Spermine (SPM), a polyamine compound that is found ubiquitiosly in plants is involved in adaptation of biotic and abiotic stresses. The effect of SPM on growth,grain filling and yield of rice at three main granaries namely, IADA BLS, MADA and KADA representing unlimited water, limited water and water stress conditions respectively, were tested during the main season. Additinally, the growth enhancer was also tested during off season at KADA. Spermine increased plant height, number of tillers per hill and chlorophyll content in all three granaries. Application of SPM improved yield by 38, 29 and 20% in MADA, KADA and IADA BLS, respectively. Harvest index showed 2.6, 6 and 16% increases at IADA BLS, KADA and MADA, respectively in SPM treated plants as compared to untreated. Except for KADA which showed a reduction in yield at 2.54 tha^−1^, SPM improved yield at MADA, 7.21 tha^−1^ and IADA BLS, 9.13 tha^−1^ as compared to the average yield at these respective granaries. In the second trial, SPM increased the yield to 7.0 and 6.4 tha^−1^ during main and off seasons, respectively, indicating that it was significantly higher than control and the average yield reported by KADA. The yield of SPM treatments improved by 25 and 33% with an increment of farmer’s income at main and off seasons, respectively. Stomatal width was significantly higher than control at 11.89 µm. In conclusion, irrespective of the tested granaries and rice variety, spermine mediated plots displayed increment in grain yield.

## Introduction

Rice (*Oryza sativa* L.), the staple food of half the human population is a cereal crop that is susceptible to soil water deficit, which causes huge yield losses in many Asian countries^1,2^. Plants adapt to drought stress or water shortage by altering physiological, biochemical and molecular processes^3,4^. Severe stress such as drought leads to significant difference in actual and potential rice yield. Tuong and Bouman^5^ predicted that by 2025, 15–20 million hectares of irrigated terrain will endure water shortage resulting in water stress that can affect the development and yield of rice^6,7^ especially if it occurs during grain filling. As a result, the farmers income and local stockpiles of rice will decrease and in fact becomes worsened in areas where rice cultivation is an annual event. When drought prevails, there are no immediate solutions that can be offered to the farmers. As a consequence, the farmers suffer from less profit and or total losses to their farming venture.

The irrigation system in major granary areas in Malaysia depends mostly on rainwater. During the main-season (August-February), paddy is mainly grown using rainfed system whereas in off-season (March to July), planting normally depends on irrigation system which might lead to water stress if it is insufficient^8^. The water supply in rainfed areas principally comes from rainfall, therefore any uncertainty in the timing of the rainfall and variability in its intensity as well as distribution, causes either flood or drought stress in rainfed lowland rice production ecosystem^9^. The percentage of losses in paddy production from 2010 to 2012 is between 22 to 14% primarily due to drought during the off season (Ministry of Agriculture and Agro-based Industries, Malaysia, 2013).

Several measures are available to mitigate the impact of drought on rice which include improved irrigation facilities in all granaries, breeding programme and simple agronomic manipulation**.** The major drawback of rice breeding program, a long-term approach, is the inability to increase productivity under normal growing conditions. Undoubtedly, managing rice under limited water availability is highly desired to ensure crop survival while enhancing yield in general for the given situation. Foliar application of growth enhancer such as polyamines that can be economical, feasible, easy to apply and readily available to the farmers could be a possible solution for rice growing in water stress environment^10,11^.

Low molecular weight aliphatic polyamines (PAs) that are present in plant cells are fundamental ubiquitous compounds for they guard and control diverse cellular functions and processes in response to biotic and abiotic stresses^12–14^. The three major forms of PAs, namely, diamine putrescine (PUT), triamine spermidine (SPD), and tetraamine spermine (SPM) mainly exist in a free, soluble conjugated or insoluble bound form. PAs play pivotal role and are central to seed germination, organogenesis, embryogenesis, budding, floral development, leaf and fruit senescence, fruit development and ripening, and plant stress responses^15–17^. However, the prominent accumulation of PAs takes place under abiotic stress such as salinity, drought, chilling, heat, hypoxia, ozone, UV-B and UV-C, heavy metal toxicity, mechanical wounding and herbicide treatment^13,16^. Prior research agree that the exogenous application of the many forms of PA at variable concentrations has conferred the plants with enhanced tolerance to various stresses ^18^ besides regulating stomatal responses by reducing their aperture and prompting its closure under water deficit conditions as a vital drought tolerant mechanism^19–21^.

Spermine, also a phytohormone, improved plants subjected to water deficit by increased dry matter yield and net photosynthesis that was associated with the maintenance of leaf water status,membrane properties and improved water use efficiency in rice^10,22^. It was also involved in different cellular processes ranging from growth promotion and cell division to inhibition of ethylene production and senescence^22^. Meanwhile, Yamaguchi et al.^23^ found that an *Arabidopsis* mutant plant, which cannot produce SPM, is hypersensitive to drought and that this phenotype was cured by SPM pretreatment.

At present, the roles of spermine on local rice varieties in relation to grain filling and yield of rice under various water regimes are not widely examined. Furthermore, earlier findings were mainly reported from the studies under normal conditions only^24–26^. Therefore, the main objective of the study is to validate the efficacy of spermine to different variabilities (soil type, rice variety, plant establishment) that prevailed during the growth cycle of rice plants at different locations, planting seasons and prevailing climate under field conditions.

## Materials and methods

To establish the bio-environmental based performance of the growth enhancer, two experiments were conducted in different granary areas and planting seasons. The experimental trials were carried out as follows:

1. Effects of spermine on growth and yield of rice at three main granary areas [Muda Agricultural Development Authority, (MADA), Kemubu Agricultural Development Authority (KADA) and Integrated Agricultural Development Authority Barat Laut Selangor (IADA BLS)] during main planting season (field trial 1).
2. Effects of spermine on yield of rice during the main and the off planting seasons at Kemubu Agricultural Development Authority (KADA), Kelantan (field trial 2).

### Field trial 1

#### Field trial and soil properties

**Figure 1:**
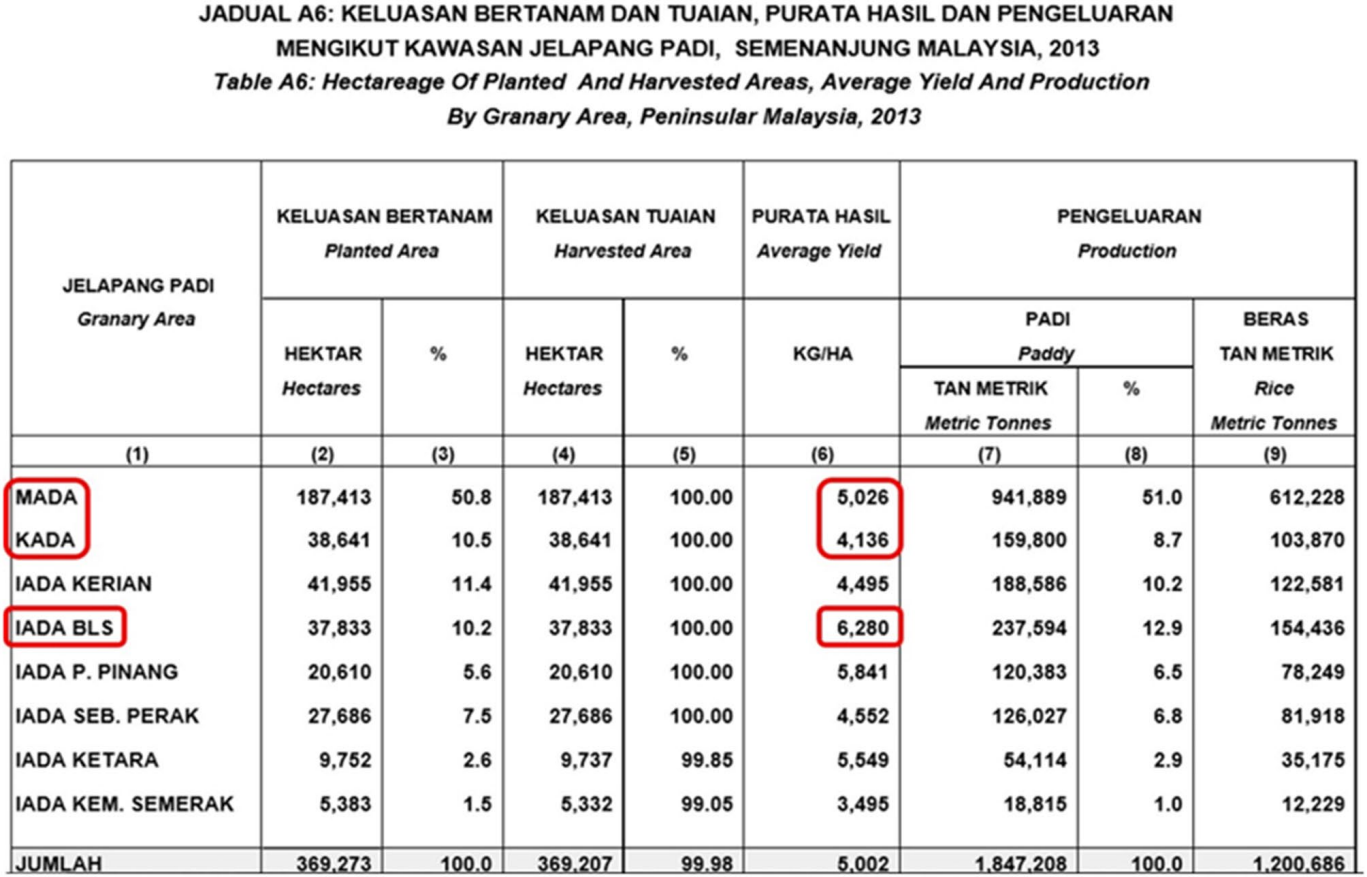
Average yield by granary area in Peninsular Malaysia in 2013.

The field trial experiment was conducted in different rice granary areas from November 2013 to April 2014. The locations of the study were at the rice fields of Kampung Hutan Buloh, Peringat in the Kemubu Agricultural Development Authority (KADA), Kelantan (5° 58’ N 102° 18’ E, 34 m elevation), Kampung Padang Teluk Mukim Jeram, Tunjang, Jitra, in the Muda Agricultural Development Authority (MADA), Kedah (6° 16’ N 100° 21’ E, 76 m elevation) and Parit 4, Sungai Burung, Sekinchan, in the Integrated Agriculture Development Area Barat Laut Selangor (IADA BLS), Selangor (3° 38’ N 101° 12’ E, 98 m elevation). The properties of soil collected from the upper 20 cm at each field were analyzed. These three locations were selected on the basis of medium, low-medium and high rice productivity (Figure 1) as indicated in granary areas productivity report according to MOA (2013). The soil properties were analyzed for physical properties (sand, silt, clay and texture classes) according to methods by Gee and Bauder^27^ and chemical analysis, namely total nitrogen, available phosphorus and available potassium according to methods of Kjeldahl, Bray and Kurtz^28^ and Metson^29^, respectively.

#### Experimental design and statistical analysis

**Figure 2:**
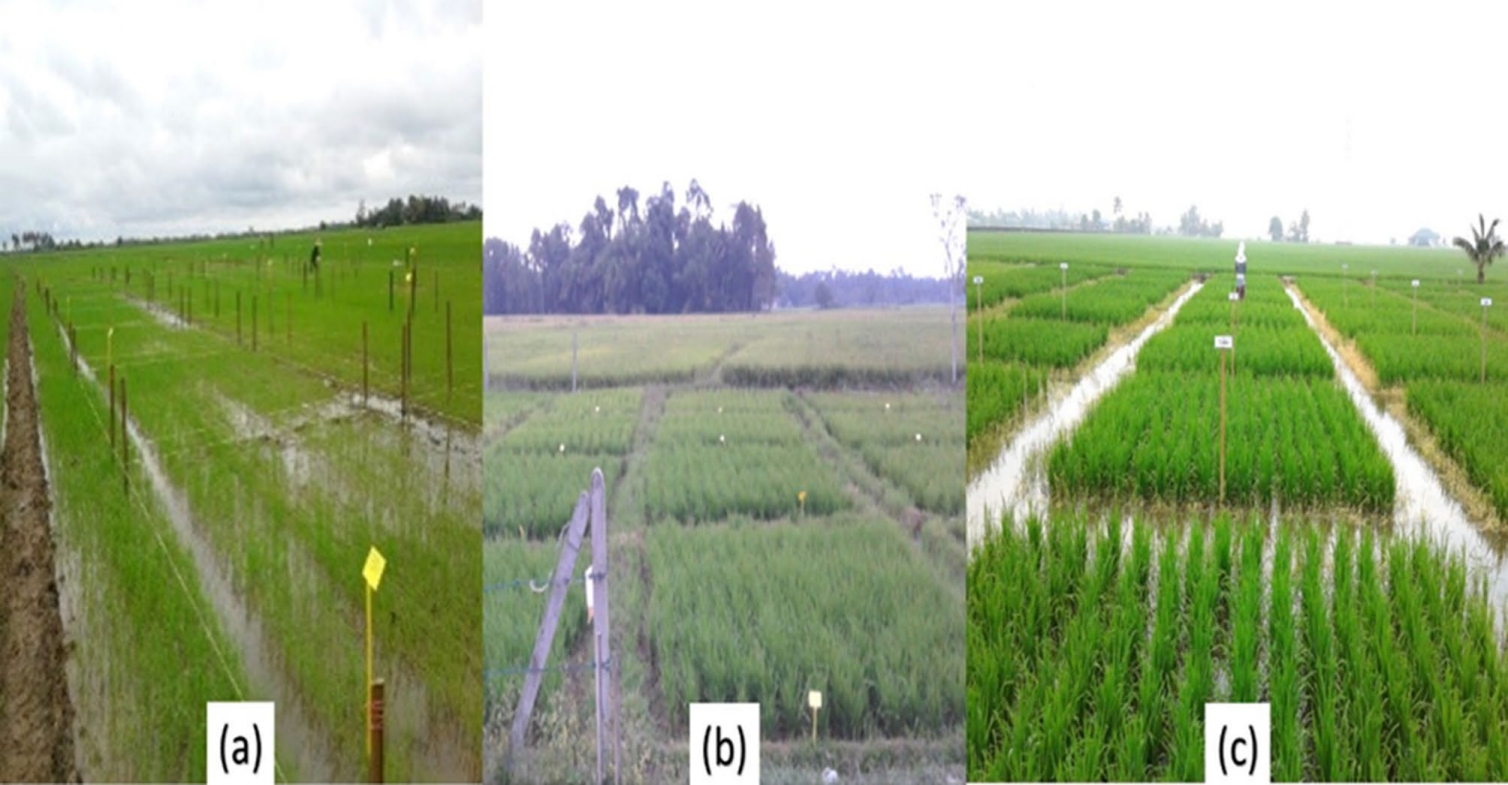
Experimental plots at (**a**) MADA, (**b**) KADA and (**c**) IADA BLS granaries.

In the field trials, foliar application of water (control) and spermine (SPM) were arranged in a Randomized Complete Block Design (RCBD) with four replications. Four plots for each experiment with 16 m^2^ (4 × 4 m) were prepared and separated by a 0.5 m boundary (approximately 16 hills per meter squared) in each of the granary areas (Fig. 2). Instead of multi-location analysis, the results were analysed based on the individual granary areas due to the following reasons;

1. Different rice varieties were grown by farmers in different granaries.
2. Method of plant establishment was different in each of the granary areas.
3. Different cultivation practices in each of the granary areas.
4. Effect of spermine was to be tested under *in-situ* conditions of the granaries to replicate the farmers’ practices.
5. Physio-chemical properties of soil at each granary was different.

#### Plant materials and cultivation

The seed variety, method of plant establishment and different cultivation practices in each of the granary areas were as follows:

**Table:** 

Granary Area	Seed Variety	Method of Plant Establishment	Cultivation Practice
MADA	MR219	Direct seeding	148 kg rice seeds per hectare
KADA	MR219	Transplanting	One seedling per hill (spacing 20 × 15 cm)
IADA BLS	MR263	Transplanting	One seedling per hill (spacing 20 × 15 cm)

*Field was thoroughly prepared and leveled before seeding and transplanting. The variety used by the farmers depended on current season to avoid weedy rice.

The soil in the experimental plot was fertilized with NPK compound fertilizer at the rate of 0.58 kgplot^−1^ (360 kgha^−1^), 0.28 kgplot^−1^ (175 kgha^−1^) and 0.28 kgplot^−1^ at 15, 50 and 70 DAS; urea was top-dressed at rate of 0.16 kgplot^−1^ (100 kgha^−1^) at 35 DAS. The field was kept flooded from transplanting until 15 days before maturity when the field was drained except at KADA trial plots that experienced water deficit during the planting season.

#### Foliar application

The control treatment was prepared by adding distilled water and 1% of Tween 20 (Sigma-Aldrich). Stock solution of SPM (Sigma-Aldarich, Malaysia) was prepared by dissolving 1 g of SPM in 100 ml of water. It was then diluted with distilled water to reach a final concentration of 70 µM of SPM according to the method described by Farooq et al.^10^. Each of the treatments was added with 1% Tween 20 (Sigma-Aldrich) as a surfactant. These solutions were stored at 5 °C before the time of application. Foliar application of SPM was carried out at 35 and 55 days after transplanting by spraying the plants uniformly to the point of run-off (approximately 100 mL m^−2^) using electric knackpack sprayer (Preco, Malaysia) for plot size 4 × 4 m^2^. Each plant in the pots was sprayed until all leaves were wet. The treatments were applied between 9.00 to 11.00 am on a clear sky day.

#### Plant height, tiller number and leaf area

Plant height was measured at 45 and 65 DAS according to methods described by Yoshida^30^ where measurement was made from the plant base to the tip of the highest leaf blade. Tiller number per hill was counted by fully expended tiller. Four hills per pot were taken randomly and measured at 45 and 65 days after sowing (DAS). Chlorophyll content of the leaves was measured by the indirect method using Portable Minolta SPAD 502 Plus (Delta T, UK) chlorophyll meter. The third fully expended leaf from top was chosen for data measurements at 45 and 65 DAS. Three replicates were taken for each treatment.

#### Microclimatic data

**Figure 3:**
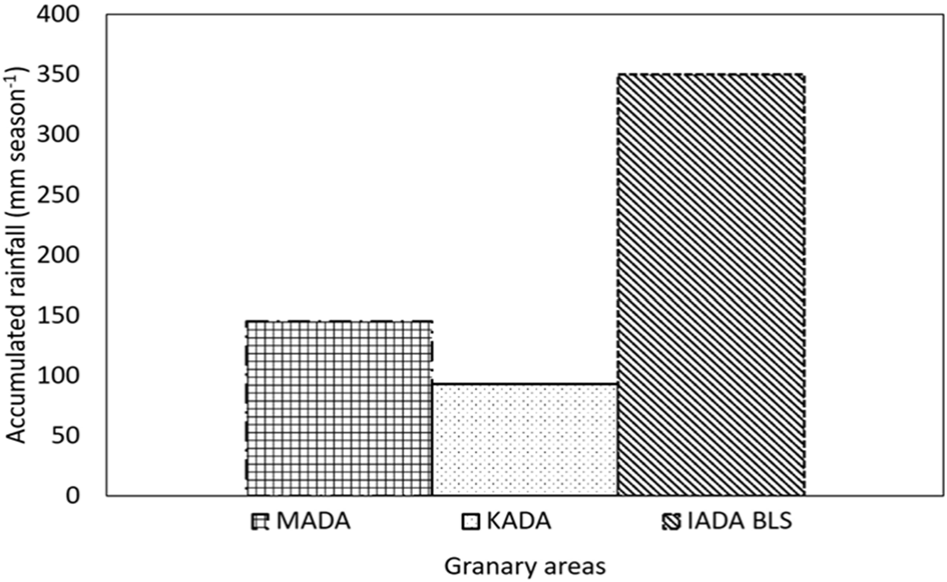
Accumulated rainfall recorded in different granaries. MADA: Muda Agricultural Development Authority, KADA: Kemubu Agricultural Development Authority and IADA BLS: Integrated Agriculture Development Area Barat Laut Selangor.

The rainfall data as shown in the Fig. 3 was obtained from Department of Meteorology, Malaysia.

#### Grain yield and yield components

The plants were harvested at maturity within a quadrat of 1 m^2^ for yield components determination. For biomass determination, the plants were separated into straw and panicles and then dried at 70 °C to for dry weight determination. The panicles were hand-threshed and filled spikelets were separated from unfilled spikelets manually. Filled and unfilled grains were counted and weighed to determine three yield components (the number of spikelets per panicle, the filled grain percentage and the 1000 grain weight)^30^.

#### Statistical analysis

All data were statistically analysed using SAS software (Windows version 9.1, SAS Institute, Cary, NC, USA). Two-sample T-test was used to test significant differences between treatments at each granary. Additionally, a one-way ANOVA was done on yield components of the three granaries for comparison. The differences among treatments were determined using the least significant difference (LSD) test at 0.05 probability level.

### Field Experiment 2

#### Field experiments and soil properties

Second field trial experiment was conducted at the National Key Economic Areas (NKEA) 11, Tegayong, KADA, Pasir Puteh Province, Kelantan (5.95 N, 102.31 E, 59 m elevation) with farmers participatory research during main-season and off-season in 2014/2015 (September–December and February-May). This granary area was selected due to lower yield (average yield 4.9 tha^−1^) as a direct consequence of low rainfall (< 200 mm) as recorded by Meteorogical Department Malaysia. The properties of soil collected from the upper 20 cm at each field were analyzed. The soil properties were analyzed for physical properties (sand, silt, clay and texture classes) according to methods by Gee and Bauder^27^ and chemical analysis, namely total nitrogen, available phosphorus and available potassium according to methods of Kjeldahl, Bray and Kurtz^28^ and Metson^29^, respectively.

#### Experimental design and statistical analysis

The second field trial was conducted in two seasons continuously with two treatments that comprised of control and spermine (SPM). Treatments were arranged in a Randomized Complete Block Design (RCBD) with three replications. The results were analysed using Statistical Analysis System (Windows version 9.1, SAS Institute, Cary, NC, USA) by T-test at P ≤ 0.05.

#### Plant materials and cultivation

Sixty kilogram of MR219 rice seeds per acre were used in both seasons for direct seeding. Field was thoroughly prepared and leveled before seeding. The farmer’s plot sizes were 0.5, 1.0 and 1.5 acres which were used as replication with farmer’s participatory research. The soil in the experimental plot was fertilized with NPK compound fertilizer at the rate of 146 kgacre^−1^ (360 kgha^−1^), 71 kgacre^−1^ (175 kgha^−1^) and 71 kgacre^−1^ at 15, 50 and 70 DAS; urea was top-dressed at rate of 41 kgacre^−1^ (100 kgha^−1^) at 35 DAS.

#### Foliar application

Foliar application of SPM was carried out at 35 and 55 days after transplanting. The SPM spray was applied until run-off with an equivalent volume of 56 L/acre by using 14 L mist blower sprayer (a Stihl SR420, USA, with 4 tanks blower per acres) in each of the plots.

#### Microclimatic data

**Figure 4:**
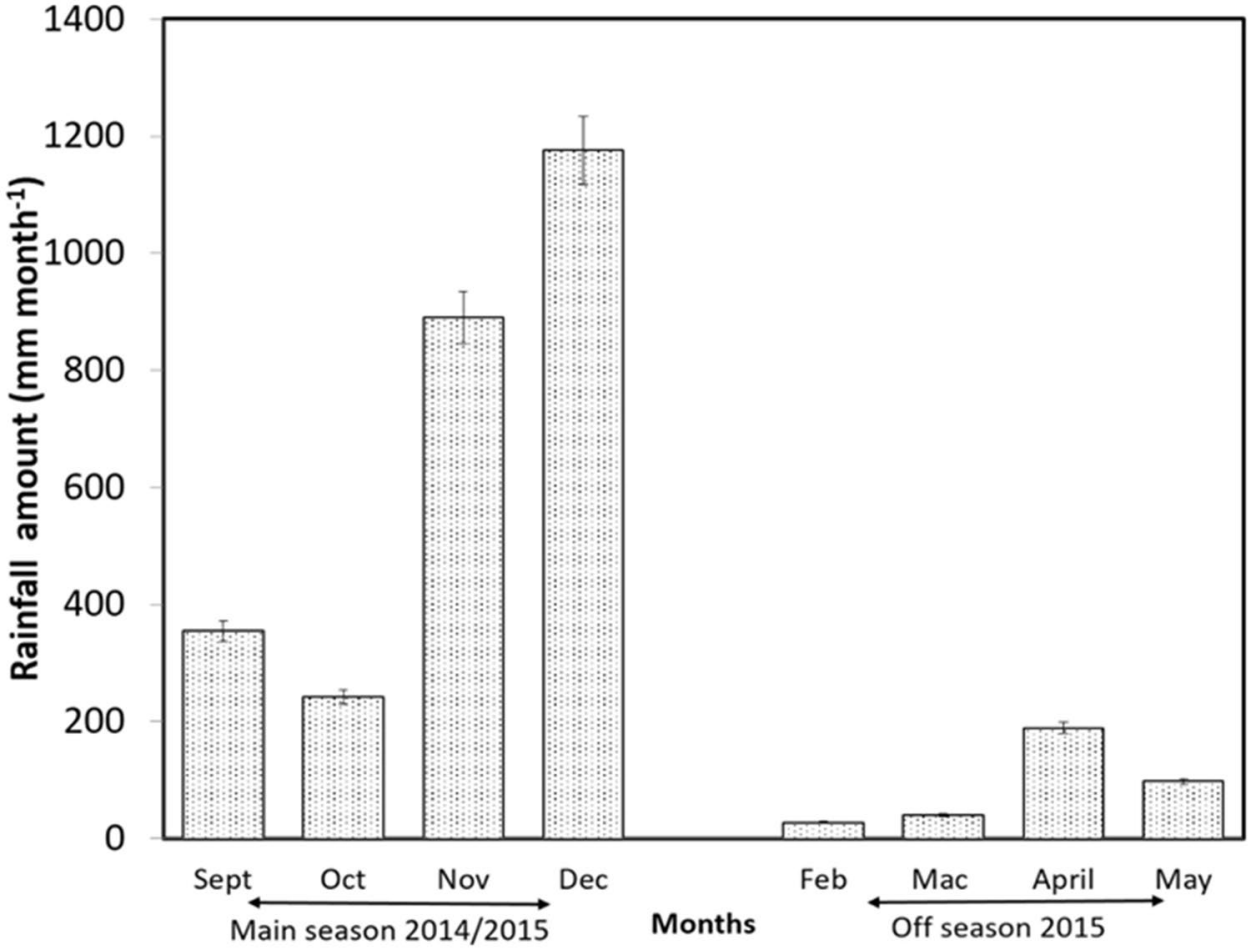
Records of monthly rainfall at KADA during main season 2014/2015 and off season 2015.

Figure 4 shows the monthly rainfall during main and off season at KADA from September 2014 until May 2015 from Department of Meteorology, Malaysia. The highest monthly rainfall ranged between 889 to 1175 mm in main season (November and December 2014). There was very little rainfall during off season at the beginning of the rice season during February, March and May 2015. Off season was relatively dry compared to main season (< 200 mm rainfall), (Malaysian Meteorology Department, 2015). The lowest rainfall recorded at off season at KADA area (total rainfall 27.4 mm) during first month of rice cultivation (February).

#### Yield

After 80% grain maturity, all plants were harvested whereby yield was determined with three replications for each treatment at the end of the experiment (120 DAS). The yield was separately harvested by a harvester machine, filled in separate lorries and weighed in the rice mills (Padiberas Nasional Berhad or BERNAS Sdn Bhd). The revenue from rice yield was calculated by multiplying rice yield (t ha^−1^) with farm-gate price (RM 1,200.00 ton^−1^). Moreover, the Malaysian government gives an additional incentive for every 1 metric tonne increase in rice yield compared with usual yield per hectare^31^. To determine the economic feasibility of SPM foliar spray, benefit cost analysis and net present revenue were carried out. Benefit–cost ratio (BCR) was calculated using the method of Nwaobiala and Adesope^32^ whereas, net present value (NPV) was determined as described by Ingabire et al.^33^.:

$$\text{Benefit-cost ratio }\left({\text{BCR}}\right) = \frac{\text{TC}}{{\text{TR}}}$$

$$\text{Net present value }\left({\text{NPV}}\right)= \sum_{\text{t=1}}^{\text{n}}\frac{{\text{(TR} \, - \, \text{TC)}}_{{\rm t}}}{{(1 \, + \, {\text{r}})}^{{\rm t}}}$$

where TC = total cost, TR = total revenue, and *r* = discount rate of rice field for per season (t). If NR or NPV > 0, then the total revenue is greater than the total cost, if NR or NPV = 0, than the total revenue is equal to the total cost, and if the NR or NPV < 0, than the total revenue is less than the total cost. In this study, NR and NPV are measured in Malaysian Ringgit (RM) and is based on one hectare. If BCR > 1, then the total revenue is greater than the total cost, if BCR = 1, than the gross revenue is equal to the total cost, and if the BCR < 1, than the gross revenue is less than the total cost.

#### Stomatal characteristics

Stomatal density,width and length was observed from the abaxial leaf surfaces. Leaves were cut into 1cmx1cm, fixed in glutaraldehyde, washed thrice in sodium cacodylate buffer and fixed in osmium tetroxide for two hours. They were then washed thrice before the serial dehydration in 35, 50, 75 and 95% acetone for half an hour at each serial dilution. In the final step, leaves were dehydrated thrice in 100% acetone for an hour. Next, the leaf segments were mounted on aluminium stubs and critical point dried before gold coating in a sputter coater. Finally, scanning electron microscope (JEOL JSM 6400) was used to view the samples at high vacuum acceleration voltage of 15 kV with a working distance of 20 mm.

### Statement of consent

This study on rice comply with relevant institutional, national, and international guidelines and legislation.

### Informed consent

Permission was obtained from Malaysian Agricultural Research and Development Institute (MARDI) to use the rice seeds for this research.

## Results

### Field experiment 1

#### Soil properties

The physico-chemical properties of the soil in the different granaries are listed in Table 1. The highest pH, organic matter, total carbon, potassium and phosphorus were 5.59, 10.32%, 0.41%, 59.2 mg kg^−1^ and 306.4 mg kg^−1^ at IADA BLS and the lowest were 10.6 mg kg^−1^ (MADA) and 67 mg kg^−1^ (KADA), respectively. In term of textural class, KADA and MADA were classified as clay loam soil while IADA BLS as silty clay soil.

**Table 1:** Physico-chemical properties of the soil used for each field trial.

Granary/determination	Physical analysis				Chemical analysis				
	Sand (%)	Silt (%)	Clay (%)	Textural classes (USDA)	pH	OM (%)	N (%)	P (mg kg^−1^)	K (mg kg^−1^)
KADA	20.2	46.7	33.0	Clay loam	5.3	2.1	0.21	36.0	67
MADA	22.9	49.1	28.0	Clay loam	5.3	2.0	0.16	10.6	121
IADA BLS	5.8	51.2	43.0	Silty clay	5.6	10.3	0.41	59.2	306

MADA: Muda Agricultural Development Authority, KADA: Kemubu Agricultural Development Authority, IADA BLS: Integrated Agriculture Development Area Barat Laut Selangor, OM: Organic matter, N: Total nitrogen, P: Available phosphorus and K: Available potassium.

#### Growth Parameter

##### MADA, Kedah

Spermine treated rice consistently had significantly higher values in growth measurement parameters compared to control plants (Table 2), except for chlorophyll content at 65 DAS. Spermine sharply increased plant height, tiller number per hill and chlorophyll content over the course of growth and development with the highest mean value of 89.8 cm, 22 tillers and 38.8 SPAD unit at 65 DAS. Control plants had obviously reduced tiller number per hill and chlorophyll content by 23 and 12% at 65 DAS, respectively compared to SPM treated plants.

**Table 2:** Effect of spermine on plant height, tiller number and chlorophyll content in MADA at different days after sowing.

Treatments	Plant height (cm)		Tiller number		Chlorophyll (SPAD value)	
DAS	45	65	45	65	45	65
Control	74	85	5	17	37.0	34.1
SPM	77*	90*	7*	22*	37.6*	38.8*
T-test	−2.89	−6.53	−5.20	−5.89	−1.79	−7.89
CV (%)	1.63	1.30	7.10	5.61	1.32	2.37

T-test was performed at P ≤ 0.05. *: Significant at P ≤ 0.05. DAS: Days after sowing, SPM: spermine, MADA: Muda Agricultural Development Authority and CV: Coefficient of variation.

##### KADA, Kelantan

Plant height and tiller number per hill at both DAT were significantly (P ≤ 0.05) higher in SPM treatments as compared to control. In addition, chlorophyll content of SPM showed an increase of 8% at 65 DAT as compared to control. A slight difference in plant height and tiller number per hill in both treatments at 45 and 65 DAT were observed (Table 3). It might be due to water stress as a result of lower rainfall recorded during that period.

**Table 3:** Effect of spermine on plant height, tiller number and chlorophyll content in KADA at different days after sowing.

Treatments	Plant height (cm)		Tiller number		Chlorophyll (SPAD value)	
DAT	45	65	45	65	45	65
Control	41	45	44	44	29.7	35.1
SPM	48*	52*	56*	55*	33.5*	38.2*
T-test	−5.71	−4.67	−3.07	−4.77	−5.55	−3.05
CV (%)	3.74	4.71	11.1	6.72	3.02	4.02

T-test was performed at P ≤ 0.05. *: Significant at P ≤ 0.05. DAT: Days after transplanting, SPM: spermine, KADA: Kemubu Agricultural Development Authority and CV: Coefficient of variation.

##### IADA BLS, Selangor

Highly significant differences (P ≤ 0.05) were observed in plant height, tiller number per hill and chlorophyll content at 45 and 65 DAT in SPM treated plants compared to control (Table 4) at IADA BLS. Similarly, chlorophyll content of SPM treated plants increased by 17 and 8% at 45 and 65 DAT, respectively.

**Table 4:** Effect of spermine on plant height, tiller number and chlorophyll content in IADA BLS at different days after transplanting.

Treatments	Plant height (cm)		Tiller number (hill^−1^)		Chlorophyll (SPAD value)	
DAT	45	65	45	65	45	65
Control	52.5	74.6	50	172	28.6	32.5
SPM	54.3*	76.7*	55*	182*	34.3*	38.3*
T-test	5.75	7.27	4.28	9.39	11.95	17.26
CV (%)	0.84	0.53	2.99	0.89	2.14	1.35

T-test was performed at P ≤ 0.05. *: Significant at P ≤ 0.05. DAT: Days after transplanting, IADA BLS: Integrated Agricultural Development Authority Barat Laut Selangor and CV: Coefficient of variation.

#### Yield components

##### MADA, Kedah

Table 5 shows the yield components between control and SPM treated plants were significantly different (P ≤ 0.05) in MADA, Kedah. Spermine treatments improved yield components by 20, 22, 23, 17, 28 and 38% in panicle length, grain number per panicle, grain filling, thousand grain weight, biomass and yield of rice, respectively compared to control. Interestingly, harvest index of SPM was greater than control at 0.58 compared to 0.50, respectively.

**Table 5:** Effect of spermine on panicle length, grain number, grain filling, thousand grain weight, biomass and yield in MADA.

Treatments	Panicle length (cm)	Grain number (panicle^−1^)	Grain filling (%)	Thousand grain weight (g)	Biomass (g m^−2^)	Yield (g m^−2^)
Control	22.2	111	70.7	23.4	895	449
SPM	27.6*	142*	91.3*	28.3*	1236*	721*
T-test	−11.27	−4.63	−12.84	−5.84	−5.11	−3.67
CV (%)	2.71	7.52	2.80	4.64	8.86	8.56

T-test was performed at P ≤ 0.05. *: Significant at P ≤ 0.05. MADA: Muda Agricultural Development Authority. CV: Coefficient of variation.

##### KADA, Kelantan

Yield components showed significant differences between control and SPM treated plants at KADA, Kelantan (Table 6). Interestingly, yield production of rice increased in SPM treated plants by 29%. Similarly, other yield components also contributed to better yield productions in SPM treatments including highest harvest index with 0.53. The grain filling percentage was increased by 19% with the application of SPM treatments. However, lowest grain filling in control (46.6%) and also SPM (57.6%) treatments were recorded as compared to normal conditions. This result might be correlated to water stress as the monthly rainfall recorded was lower than 200 mm. Following that, more empty grains were produced compared to filled grains that directly contributes to reduction of yield production (1.8 and 2.54 tha^−1^) in both treatments compared to the average yield reported by KADA, 2013 (4.0 tha^−1^). Harvest index showed 6% increment in SPM treated plants as compared to control at 0.50.

**Table 6:** Effect of spermine on panicle length, grain number, grain filling, thousand grain weight, biomass and yield in KADA.

Treatments	Panicle length (cm)	Grain number (panicle^−1^)	Grain filling (%)	Thousand grain weight (g)	Biomass (g m^−2^)	Yield (g m^−2^)
Control	22.5	113	46.6	23.5	365	181
SPM	25.8*	132*	57.4*	25.2*	480*	254*
T-test	−6.08	−3.89	−4.68	−8.31	−7.67	−3.37
CV (%)	3.20	5.42	6.28	1.84	5.02	6.06

T-test was performed at P ≤ 0.05. *: Significant at P ≤ 0.05. KADA: Kemubu Agricultural Development Authority. CV: Coefficient of variation.

##### IADA BLS, Selangor

Table 7 shows spermine treated plants significantly enhanced yield productions indicating that yield components also provided positive effects at IADA BLS, Selangor. The grain number, grain filling, thousand grain weight, biomass and yield production improved by 24, 8, 7, 18 and 20% in SPM treatments, respectively compared to control. Both treatments increased yield production of rice by 7.3 and 9.1 tha^−1^ compared to average yield of 6.3 tha^−1^ in year 2013 as reported by DOA. Harvest index in SPM and control treatments were 1.16 and 1.19 respectively.

**Table 7:** Effect of spermine on panicle length, grain number, grain filling, thousand grain weight, biomass and yield in IADA BLS.

Treatments	Panicle length (cm)	Grain number (panicle^−1^)	Grain filling (%)	Thousand grain weight (g)	Biomass (g m^−2^)	Yield (g m^−2^)
Control	22.2	121	77.9	24.6	630	729
SPM	27.1*	159*	84.9*	26.5*	770*	913*
T-test	−21.64	−31.95	−17.78	−5.20	−10.84	−19.08
CV (%)	1.30	1.19	0.68	2.05	2.61	1.02

T-test was performed at P ≤ 0.05. *: Significant at P ≤ 0.05. IADA BLS: Integrated Agricultural Development Authority Barat Laut Selangor. CV: Coefficient of variation.

##### Yield components at all three granaries

All the yield components, namely grain number, 1000 grain weight, grain filling percentage, biomass and yield showed significant interaction between treatment and granary (Table 8). Both factors of treatment and granary were significantly different too. Spermine improved yield paramaters compared to control. Meanwhile, in general, the yield components in IADA and MADA showed better performance compared to KADA.

**Table 8:** Main and interaction effects of treatment and granary on yield components of rice variety.

Factor	Panicle Length (cm)	Grain number	1000 Grain Weight (g)	Grain Filling (%)	Biomass (g/m^2^)	Yield (g/m^2^)
**Treatment**						
Control	22.3 b	115 b	23.80 b	65.05 b	629.93b	452.92 b
Spermine	26.8 a	144 a	26.68 a	77.85 a	828.58 a	629.33 a
Pr > F	***	***	***	***	***	***
**Granary**						
IADA	24.6 ab	140 a	25.54 a	81.39 a	700.00 b	820.88 a
KADA	24.1 a	122 b	24.33 b	51.95 b	422.50 c	217.50 c
MADA	24.9 a	126 b	25.85 a	81.01 a	1065.26 a	585.00 b
Pr > F	*	***	*	***	***	***
Treatment × Granary	**	*	**	***	**	***

Means followed by the same letter in the same column are not significantly different at p ≤ 0.05 according to Fisher’s least significant difference (LSD).ns indicates non-significant difference at P > 0.05, * significant difference at P ≤ 0.05, ** significant difference at P ≤ 0.01 and *** significant difference P ≤ 0.001. IADA BLS: Integrated Agricultural Development Authority Barat Laut Selangor, KADA: Kemubu Agricultural Development Authority, MADA: Muda Agricultural Development Authority.

Thousand grain weight, grain filling percentage, grain number, biomass and yield were very highly significant (Table 9). Spermine treated plants in MADA had the heaviest 1000 grain weight followed by SPM treated plants in IADA and KADA whereas the control treatment at all three granaries were not significantly different among one another. As for grain filling, once again SPM treated plants in MADA showed a significantly higher percentage followed by SPM treated plants in IADA and control plants in both IADA and MADA. Treated and untreated plants at KADA performed poorly. Meanwhile, the number of grains per panicle was highest in SPM treated plants in IADA followed by SPM treated plants in KADA and MADA. Unlike the rest of the yield components, biomass showed a very clear trend. Biomass at MADA was significantly higher than the two granaries irrespective of treatment. Similarly, biomass at IADA BLS was significantly higher than KADA. On the other hand, SPM treated plants in IADA recorded the highest yield followed by untreated at the same granary and SPM treated plants at MADA, the latter two were not significantly different from one another. Control plants at KADA recorded the lowest yield.

**Table 9:** Yield components of treatment combination.

Treatment	1000 Grain Weight (g)	Grain Filling (%)	Grain Number	Biomass (g/m^2^)	Yield (g/m^2^)
IADA Control	24.58 cd	77.90 c	121 c	630.00 d	728.50 b
IADA SPM	26.50 b	84.88 b	159 a	770.00 c	913.25 a
KADA Control	23.45 d	46.55 f.	113 cd	365.00 f.	181.25 e
KADA SPM	25.20 bc	57.35 e	132 b	480.00 e	253.75 d
MADA Control	23.38 d	70.70 d	111 d	894.78 b	449.00 c
MADA SPM	28.33 a	91.33 a	142 b	1235.75 a	721.00 b
Pr > F	***	***	***	***	***
LSD	1.43	2.22	10	88.46	20.32

Means followed by the same letter in the same column are not significantly different at p ≤ 0.05 according to Fisher’s least significant difference (LSD). *** significant difference P ≤ 0.001. IADA BLS: Integrated Agricultural Development Authority Barat Laut Selangor, KADA: Kemubu Agricultural Development Authority, MADA: Muda Agricultural Development Authority, SPM: spermine.

### Field experiment 2

#### Yield productions

**Figure 5:**
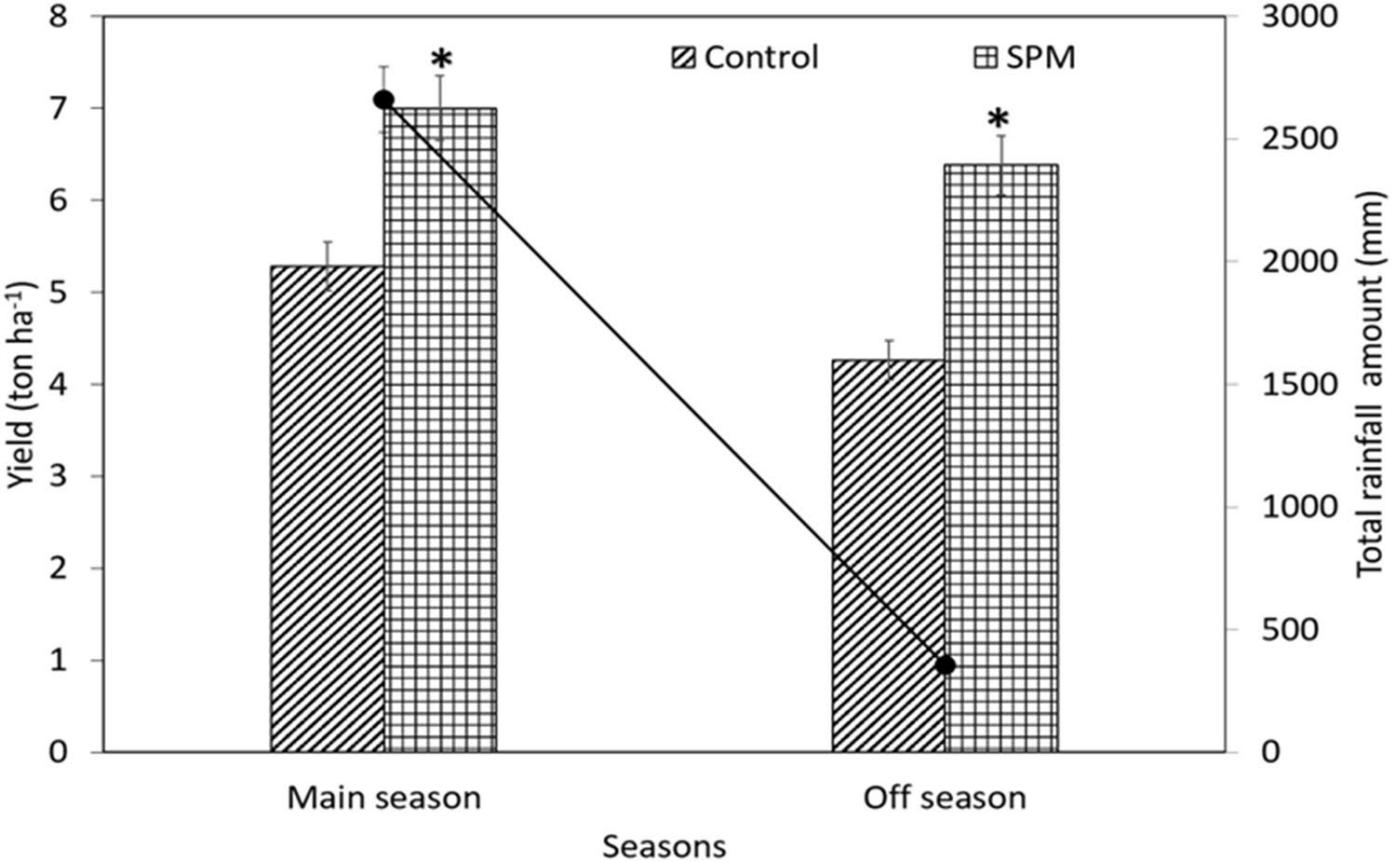
Effect of SPM treatment on yield production and monthly rainfall in rice plants during main and off seasons. Bars represent standard errors of means (n = 3). T-test was performed at P ≤ 0.05 (n = 4). *: Significant at P ≤ 0.05.

The rice yield production in control and SPM treatments during both seasons were presented in Fig. 5. The treatments from previous field experiment (main season) were used again in off season where a lower rainfall was recorded (less than 100 mm of total rainfall recorded for 3 months). Spermine foliar application treatments increased yield production to 7.0 and 6.4 tha^−1^ during main and off seasons, respectively, indicating that the yield was significantly higher than control (5.3 and 4.3 tha^−1^) and the average yield reported by KADA (2015) with 4.8 tha^−1^. The yield of SPM treatments improved by 25 and 33% with an increment of RM 1612 ha^−1^ and RM 2129 ha^−1^ in farmer’s income at main and off seasons, respectively.

#### Economic viability of adopting SPM foliar spray in rice cultivaion

Table 10 summarizes the benefit–cost ratio (BCR) and net present value (NPV) of the different treatments during main and off season. The SPM treatment was profitable during both seasons. A relatively higher rice yield was associated with SPM treatments (6.4 to 7 t ha^−1^) as compared to the national (Malaysia) average yield (4.8 t ha^−1^) in both seasons. For every RM 1 spent in adopting the SPM treatments, RM 1.60 and RM 1.62 were gained in return during main and off season, respectively. In terms of net present value, the SPM treatments more economically viable compared to control with additional income of RM 1621.71 ha^−1^ and RM 2128.98 ha^−1^ during main and off season, respectively.

**Table 10:** Effect of different planting seasons on benefit–cost ratio (BCR) and Net Present Value (NPV).

Treatment	Main season		Off season	
	BCR	NPV (RM ha^−1^)	BCR	NPV (RM ha^−1^)
Control	1.32	1455.49	1.23	1037.55
SPM	1.60	3068.20	1.62	3166.53

#### Stomatal features

**Figure 6:**
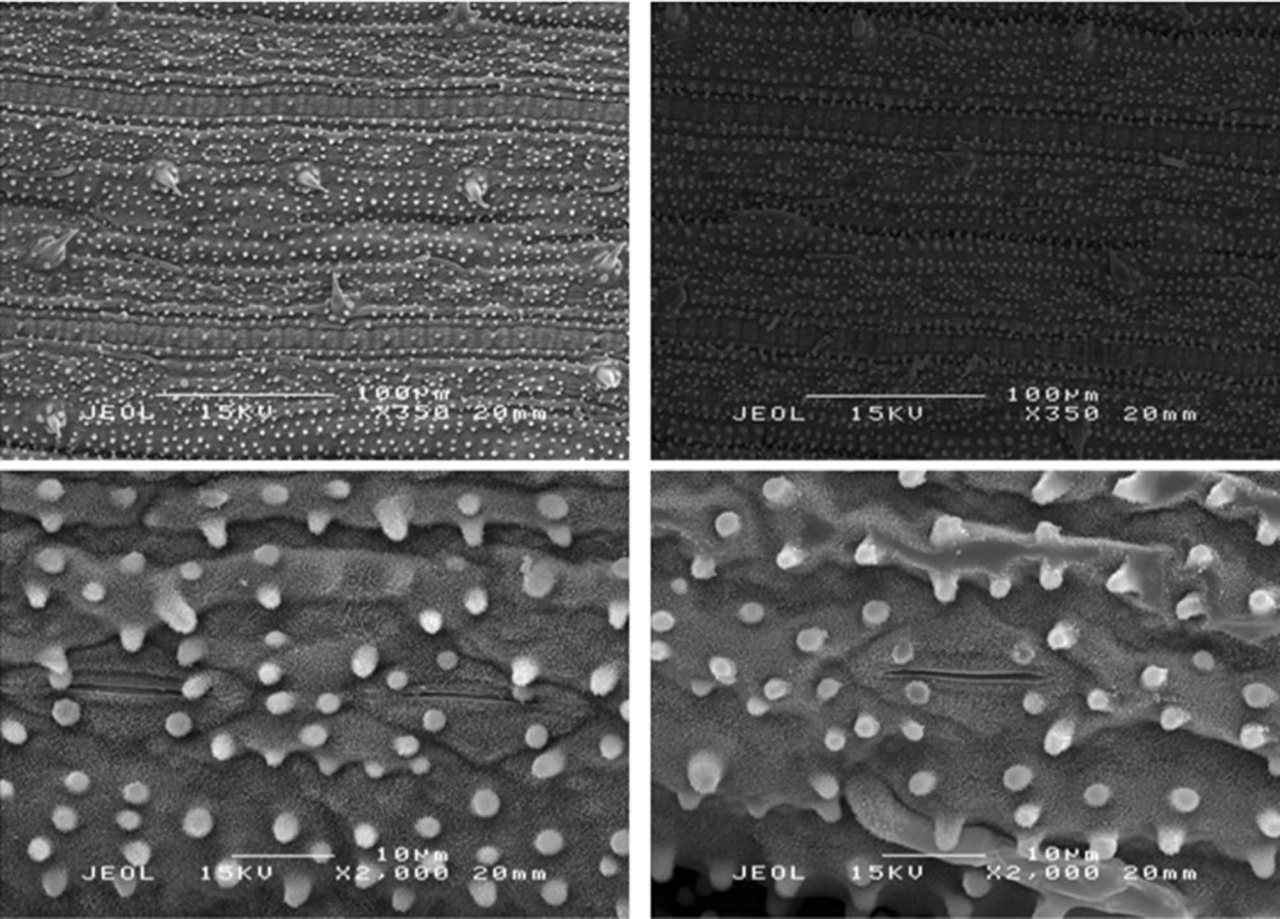
Stomatal features of control (left) and SPM treated (right) rice plants at KADA during off season.

Stomatal density was found to be higher in control plants at 43 per area though not significantly higher than SPM treatment at 39 (Table 11). Although stomatal length was found to be insignificant, nevertheless it was slightly higher in SPM compared to control. Meanwhile, stomatal width was significantly higher than control at 11.89 µm (Table 11). Figure 6 showes the stomatal apparatus of MR219 rice variety.

**Table 11:** Effect of SPM on stomatal density, length and breadth of MR219 in KADA during off season.

Treatment	Stomatal density per field of view (FOV)	Stomatal length (µm)	Stomatal width (µm)
Control	43	22.88	10.71
SPM	39	23.69	11.89*
T-test	0.58 ^ns^	1.16 ^ns^	2.48

T-test was performed at P ≤ 0.05. *: Significant at P ≤ 0.05.

## Discussions

Abiotic stress such as drought is a multifaceted stress condition that inhibits crop growth and causes serious crop yield limitations^34^. The reproductive stage of rice plant is critical and the most sensitive to water shortage which can lead to sterility of the reproductive organs which will then result in empty grains^35^ and hence can reduce grain yield by 35–75% in different cultivars of rice, which display wide genotypic differences in susceptibility to water stress during this period^36,37^.

In this study, both methods of rice plant establishment, transplanting and direct seeding were used. Generally, rice planting is dependant on about 27% of total fresh water withdrawal^37^ while puddling in wetland rice cultivation needs 30% of the total water consumption^38^.Although transplanting requires less seed, it is highly labour intensive and requires longer time to maturity due to transplanting stress^39^. Puddling which is done to create a hard pan below the plough zone had led to high losses of water. As water crisis becomes severe, this method of planting will give rise to high labour cost, thus reducing profit. Although transplanting could inprove yield as seedlings with well-developed rootlets are used, it has to be done at the optimum transplanting time, otherwise weak tillering will lead to reduced yield ^40^. Hence, in Malaysia, direct seeding method has gained much attention for its low water demand. In this method, seeds or pre-germinated seeds are directly sown in the field and as such transplanting shock is avoided. Three methods are employed in direct seeding, namely dry-seeding, wet-seeding and water-seeding^41^. The proportion of direct seeded land under rice cultivation is believed to be highest in Peninsular Malaysia amongst all the Asian countries.

Advantages of direct seeded rice are reduced labour requirement, as much as 50% depending on environment, increased water use efficiency and shorter crop rotation as a result of reduced stress^42^. Plants reach maturity stage earlier for they need not reproduce rootlets as in the case of transplanted rice. Nevertheless, direct seeding has its drawbacks too. There is greater competition between weed and rice due to same age. Hence, farmers incur extra cost to control the notorious weeds. In addition, plants tend to lodge more due to poor and shallow root anchorage as seeds are planted at shallow depth^42^. Furthermore, high plant density in direct seeded rice results in excessive stem elongation and thin stems^43^. Poor and uneven establishment and inadequate weed control are the major reasons for lower unfilled or and partially filled spikelets^44,45^. Other major problems with direct-seeded rice include difficulties in controlling snails and quality deterioration resulting from harvest that may occur during the rainy season. Seed priming is often employed to improve the germination performance and seedling establishment of direct-seeded rice ^46^. Besides, this technique could to improve speed and uniformity of seed emergence, elevate seedling vigor, and yield of cereal crops^47^. In a nutshell, both methods have their fair share of benefits and down-falls, thus it is at the discretion of the farmers to decide on the method of rice cultivation in addition to the planting material, herein rice variety.

Polyamines are endogenous plant growth regulators that mediate many plant physiological processes and the manipulation of endogenous PA metabolism or external application of PA could affect the resistance of crops to drought stress^48,49^. In the present study, SPM, a form of PA was found to have a profound effect in improving growth and yield of rice under differing water availability. Three granaries, namely, KADA, MADA and IADA BLS were selected on the basis of low, medium and high rice productivity, respectively (Fig. 1) as indicated in granary areas productivity report according to Ministry of Agriculture, Malaysia (2013) and water availability (Fig. 3). In the first field trial, application of SPM improved yield by 38, 29 and 20% in MADA, KADA and IADA BLS, respectively compared to control. The better yield in SPM treatments was mainly attributed to the higher filled grain percentage (19%) and was found to be higher than the national average yield of 4.5 t ha^−1^^50^. As soil characteristics in all granary areas differed, the difference in nutrient compositions and soil texture class resulted in different yield production of rice^51–53^ due to the initial fertility of the cultivated land^54^. Hence, both MADA and KADA recorded a higher percentage of yield increment in presence of spermine as opposed to IADA BLS for the room to maximize productivity and benefit from nutrient input is generally higher in low productivity granaries. The IADA BLS granary recorded the highest organic matters, total nitrogen and potassium followed by MADA and KADA. Kemubu Agricultural Development Authority (KADA) had the lowest total nitrogen content and available potassium content compared to other granaries. Additional nutrients in the soil enhanced plant growth, which indicates higher translocation of assimilates and partitioning of carbon reserved in stem or leaves and grains^55^.

In the current study, we opted to replicate in-situ planting conditions and thus used MR219 and MR263. Both varieties were released by Malaysian Agricultural Research and Development Institute (MARDI) which has a paddy and rice unit under its wing and does rice breeding. MR219 that was released in 2001, was the first variety developed using the direct seeding method and was a high yielding variety that used to be planted in about 90% of granaries up till a couple of years back. While the average yield is in the region of 6.5 ton ha^−1^, it could reach a maximum yield of 10.7 ton ha^−1^^56^. The maturity period was 105–111 days; the shortest life cycle compared to previously released varieties and was fairly resistant towards blast and bacterial leaf blight ^57^. As for MR263, it was released a year later to ensure the rice granaries had back up varieties with good agronomic traits in case there was a disease breakout and to complement the existing pool of rice varieties. It was also a high yielding variety with an average yield of 7.5–9 ton ha^−1^ with a shorter maturation date (97–104 days)^58^. MR263 was initially developed to be planted in moderately fertile soils and organic rice fields. To put things in perspective, any rice variety will succumb to pest and disease attack if planted for more than 20 consecutive seasons. In 2015, a total of 4,033 ha was infected by rice blast which led to 50–70% of yield losses^59^. Present day farmers are more open to a change in cultural agronomic practices, hence are more accommodative to try new rice varieties when one fails. In our trial, SPM-mediated treatment using MR263 at IADA BLS exhibited the highest yield followed by MR219 at MADA and KADA.

The IADA BLS granary is the most productive granary in Malaysia for it produces an average of 6.28 kg/ha of rice (Fig. 1). Good cultural practices, fertile soil and functional irrigation system contributes to the high yield. The soil at this granary contains the highest organic matter, a property that is associated with increased soil fertility. Organic matter is mainly made up of decomposed plant and animal residues. FAO had termed organic matter as revolving nutrient fund which could also improve soil structure and maintain tilt. According to Stevenson^60^, humus in organic matter could enhance soil water holding capacity and CEC, and aeration through good soil structure.

Besides, the plant in IADA BLS is grown in silty clay soil compared to MADA and KADA where rice is grown in clay loam soil (Table 1). The difference in these two types soil is the proportion of sand; clay loam contains a higher percentage of sand compared to silty clay. Soil texture which refers to the particle size in soil affects water holding capacity (WHC). Clay which has the smallest particle size has high WHC, thus do not drain fast while sand, made up of large particles loses water very quickly for it has low WHC. Sandy soil has poor retention of plant nutrients though it is more permeable to air, water, and roots, which are suitable for crop growth^61^. As such farmers will need to supplement with an adequate supply of nutrients.

Hudson^62^ have proven that as WHC of the soil more than doubled, the soil organic matter content increased by five-folds. Correspondingly, clay soil has a higher physical protection of soil organic matter as compared to sandy soil^63^. As soil texture affected the available water holding capacity and organic matter, clay soil positively impacted yield components by producing more tillers and grain bearing panicles, heavier seeds and higher grain filling against sandy soil.

Though both KADA and MADA share the same type of soil, the same can’t be said of its effect on yield components. Having a high percentage of sand particles in the soil will drain the soil off water and nutrients at a faster pace. While it may not be an issue at MADA which gets uninterrupted water supply, KADA which is located in a drought prone area will be unable to compensate for water loss. Lack of water at crucial stages such as at reproductive stage may lead to high percentage of grain sterility. According to Harun et al.^64^, the best technological practices are in IADA BLS followed by MADA and lowest in KADA. Tillage practices, water management, land preparation and seed preparation at IADA BLS is the best among the granaries in the country. This was evident in thousand grain weight, grain number grain filling percentage and yield of untreated plants as these parameters recorded much higher values at IADA BLS. In addition, the soil at KADA had a much lower potassium content compared to MADA which might have caused early leaf senescence leaf wilting, and leaf rolling when temperature is high and humidity is low in addition to unhealthy root system reduction in the uptake of other nutrients.

The dynamic increase of yield in three different locations with different climatic conditions and soil properties were proven by SPM foliar applications. Based on these results, a second trial was conducted at KADA, Kelantan for it was the most infertile granary as it had the lowest nutrient compositions and has a history of drought related stress as proven by the lowest rainfall received among the three granaries tested.

The experiment was conducted to evaluate the yield production, cost and income of farmers in both seasons (main and off seasons). In terms of cost, SPM treatments was very economical as if benefited the farmers with better return especially during the off season, a crucial time for farmers to survive. Eventhough the yield production was very low at off season, additional SPM foliar application still improved yield by 67% and an extra income of RM 2129 ha^−1^. Based on the main season, an increase of 42% will impact the national rice production on average yield and SSL by 5.8 tha^−1^ and 92%, respectively. Interestingly, the economic viability of adopting SPM foliar spray in rice cultivation was very practical based on increases of NPV by 53 and 67% during main and off season, respectively.

Water stress affects stomatal morphology and the results obtained in this study were in agreement with previous trials^65–67^. Water deficit increased stomatal density on the leaf area whereas stomatal length and width were reduced. Increased stomatal density in a water stress condition is often accompanied by a decrease in the volume of the cells that form the stomatal apparatus which was apparent in this study^68,69^. Study of stomatal size and density in various crops growing under water shortage revealed a negative relationship between stomatal density and stomatal size^65,70,71^. In contrast, the size of stomata was higher in SPM treated plants. The size and density of stomata varies according to species and the environment and is controlled by genetic and environmental factors^65,72,73^. As stomata plays a pivotal role in regulating photosynthesis, size and density are important ecophysiological traits controlling plant water use, especially in water-limited environments^74–77^.

Spermine improved plant height, tiller number and chlorophyll content compared to control treatments due to increased cell division and enlargement of sink size. These results indicated that more translocation of assimilates and partitioning of carbon are reserved in stem or leaves for expansion and elongation of organs^78,79^. In a previous study^80^, it was revealed that the application of SPM and with double spray generated higher stomatal conductance and photosynthesis rate. According to past researches, SPM might stimulate efficient sink activity by the metabolic activity of assimilate uptake and conversion^10,14,81^. At the same time, better dry matter partitioning to generative storage organs compared to meristematic tissues and vegetative storage organs might have occurred with the use growth enhancers.

It is obvious that the application of SPM on rice plants leads to improved plant tugor and manage to mitigate water stress by adjustment of stomatal opening and also root hydraulic conductivity^6,14,82,83^. Studies had shown that SPM enhanced photosynthesis rate, stomatal conductance^84^ and yield production^85^. Li et al.^86^ reported that SPM helped to maintain water balance under drought stress by increasing expression of the Ca^2+^-dependent AQPs, TrTIP2-1, TrTIP2-2, and TrPIP2-7. In addition, the growth enhancer can regulate several abscisic acid-related genes, which in turn control stomatal closure, stress-response gene expression and osmolyte production^87^. Meanwhile, Krishnan and Merewitz^88^ reported that exogenous SPM application increased the concentration of abscisic acid and gibberellic acid in bentgrass under drought stress. Yang et al.^89^ deduced that the yield maintenance ratio of rice was positively correlated to increasing PAs during water stress. Apparently, the biosynthesis of PA is enhanced during stress in stress-tolerant plants resulting in a two- to threefold increase of endogenous PA levels as opposed to stress intolerant plants ^90^.

## Conclusion

Double application of SPM on rice plants is an alternative way to improve plants under water stress conditions through higher grain filling. The yield of SPM foliar sprayed plants were consistently enhanced as compared to control in all granaries. It showed SPM continuosly enhanced grain filling and yield of rice in both planting seasons compared to control. Based on the benefit cost ratio and net present value, SPM treatments was very practical and economical in terms of economic return especially during off season.
